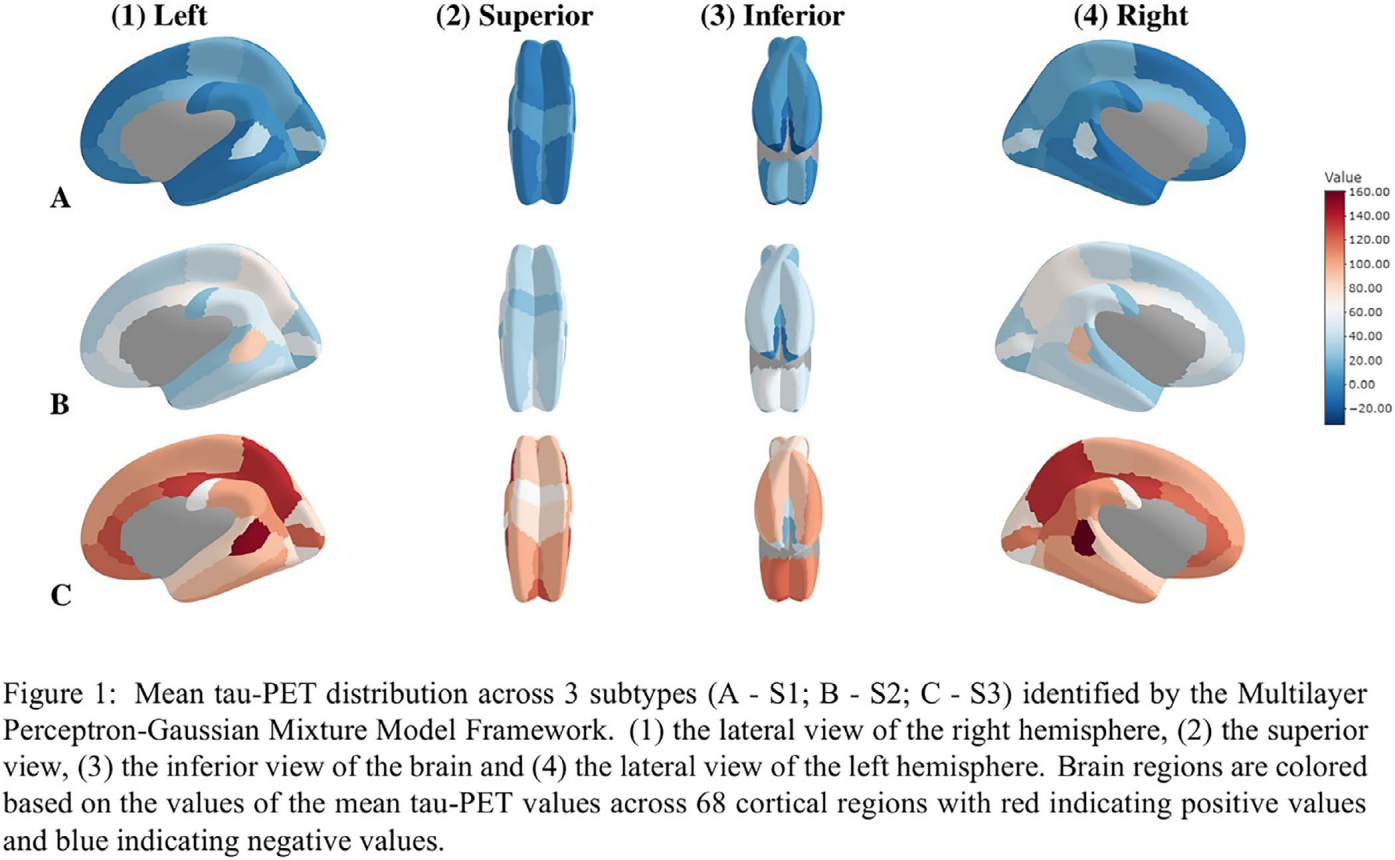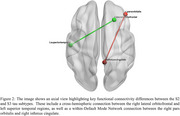# Deep Learning Framework for Characterizing Tau‐PET Spatial Heterogeneity along AD Spectrum: A Self‐supervised Approach

**DOI:** 10.1002/alz70862_109949

**Published:** 2025-12-23

**Authors:** Theyaneshwaran Jayaprakash, Connor Lee Cornelison, Plamena P. Powla, Lu Zhang, Carolyn A Fredericks, Andrew J. Saykin, Selena Wang

**Affiliations:** ^1^ Indiana University, Bloomington, IN USA; ^2^ Indiana University School of Medicine, Indianapolis, IN USA; ^3^ Indiana University Indianapolis, Indianapolis, IN USA; ^4^ Yale School of Medicine, New Haven, CT USA; ^5^ Indiana Alzheimer’s Disease Research Center, Indiana University School of Medicine, Indianapolis, IN USA; ^6^ Department of Radiology and Imaging Sciences, Indiana University School of Medicine, Indianapolis, IN USA

## Abstract

**Background:**

Extracellular amyloid plaques and intraneuronal neurofibrillary tangles are defining features of Alzheimer disease (AD). AD patients exhibit spatially heterogeneous tau‐PET composition across brain. This cross‐sectional study aims to identify distinct population subtypes along AD spectrum using novel deep learning algorithm based on tau‐PET spatial patterns.

**Method:**

We analyzed tau‐PET data from 318 (ADNI Phase 3) participants using novel self‐supervised framework with Gaussian Mixture Models (Kang et al., 2024) to identify subtypes based on tau accumulation across 68 brain regions, defined by Desikan‐Killiany atlas. Model performance was assessed using 10‐fold cross‐validation (90‐10 split ratio). Model‐estimated subtypes were validated using clinical assessments, anatomical volumetric measurements, genetics (APOE4 alleles), and functional connectomes to confirm their distinctiveness. Two‐sample t‐tests were conducted to evaluate statistical significance of differences among identified subtypes, with adjustments for multiple pairwise comparisons. Functional connectome differences were analyzed using novel Bayesian latent space model (Wang et al., 2024).

**Result:**

Three robustly differentiated AD subtypes (S1, S2, and S3) were identified, with 93% validation accuracy and Adjusted Mutual Information scores >0.89, each exhibiting distinct tau patterns. S1 showed minimal transentorhinal/temporal pole tau accumulation, S2 had increased medial temporal/posterior parietal tau binding, and S3 demonstrated widespread cortical tau accumulation (see Figure 1). Cross‐sectional analysis revealed significantly higher whole‐brain tau accumulation (TAU_bl) in S2 than S1 (*p* <0.001). Cognitive assessments revealed domain specific deficits: memory impairment (RAVLT_immediate_bl) from S1 to S2, language deficits (LDELTOTAL_bl) from S2 to S3, and more severe overall cognitive impairment (MMSE_bl) from S1 to S3. Patient‐reported measures remained stable, while study partner assessments indicated greater impairment in memory (EcogSPMem_bl), planning (EcogSPPlan_bl), and organizational (EcogSPOrgan_bl) skills in S3 than S2. Two significant functional connectivity differences were identified between S2 and S3 (see Figure 2) based on non‐overlapping 95% credible intervals: Cross Hemispheric connection (right lateral orbitofrontal to left superior temporal) and Default Mode Network connection (right pars orbitalis to right isthmus cingulate).

**Conclusion:**

This study leveraged tau‐PET data to identify spatial heterogeneity in AD. Multimodal analyses highlighted anatomical, cognitive, and functional connectivity differences between subtypes, emphasizing the potential of deep learning models to explore AD pathology.